# Quantifying the role of photoacclimation and self-facilitation for seagrass resilience to light deprivation

**DOI:** 10.3389/fpls.2023.1186538

**Published:** 2023-07-21

**Authors:** Mario Minguito-Frutos, Matthew P. Adams, Teresa Alcoverro, María P. Vilas, David Alonso, Elvira Mayol, Jaime Bernardeu-Esteller, Lázaro Marín-Guirao, Juan M. Ruiz, Jordi Boada

**Affiliations:** ^1^ Centre d’Estudis Avançats de Blanes (CEAB-CSIC), Carrer d’Accés a la cala Sant Francesc, Girona, Spain; ^2^ School of Mathematical Sciences, Queensland University of Technology, Brisbane, QLD, Australia; ^3^ Centre for Data Science, Queensland University of Technology, Brisbane, QLD, Australia; ^4^ School of Chemical Engineering, The University of Queensland, St. Lucia, QLD, Australia; ^5^ Department of Environment and Science, Queensland Government, Brisbane, QLD, Australia; ^6^ Departament de Biologia Evolutiva, Ecologia i Ciències Ambientals, Facultat de Biologia, Universitat de Barcelona, Barcelona, Spain; ^7^ Department of Global Change Research, IMEDEA (Mediterranean Institute for Advanced Studies) (UIB-CSIC), Esporles, Spain; ^8^ Seagrass Ecology Group, Oceanographic Center of Murcia, Spanish Institute of Oceanography (IEO-CSIC), Murcia, Spain; ^9^ Laboratoire d’Océanographie de Villefranche-sur-Mer, CNRS, Sorbonne Université, Villefranche sur mer, France

**Keywords:** minimum light requirements, physiological photoacclimation, bistability, resilience, *Cymodocea nodosa*

## Abstract

**Introduction:**

Light gradients are ubiquitous in marine systems as light reduces exponentially with depth. Seagrasses have a set of mechanisms that help them to cope with light stress gradients. Physiological photoacclimation and clonal integration help to maximize light capture and minimize carbon losses. These mechanisms can shape plants minimum light requirements (MLR), which establish critical thresholds for seagrass survival and help us predict ecosystem responses to the alarming reduction in light availability.

**Methods:**

Using the seagrass *Cymodocea nodosa* as a case study, we compare the MLR under different carbon model scenarios, which include photoacclimation and/or self-facilitation (based on clonal integration) and that where parameterized with values from field experiments.

**Results:**

Physiological photoacclimation conferred plants with increased tolerance to reducing light, approximately halving their MLR from 5-6% surface irradiance (SI) to ≈ 3% SI. In oligotrophic waters, this change in MLR could translate to an increase of several meters in their depth colonization limit. In addition, we show that reduced mortality rates derived from self-facilitation mechanisms (promoted by high biomass) induce bistability of seagrass meadows along the light stress gradient, leading to abrupt shifts and hysteretic behaviors at their deep limit.

**Discussion:**

The results from our models point to (i) the critical role of physiological photoacclimation in conferring greater resistance and ability to recover (i.e., resilience), to seagrasses facing light deprivation and (ii) the importance of self-facilitating reinforcing mechanisms in driving the resilience and recovery of seagrass systems exposed to severe light reduction events.

## 1 Introduction

Seagrasses provide multiple goods and services to humans, such as nursery habitat for fish species, coastal protection against erosion, water quality improvement, carbon sequestration, and buffering capacity against ocean acidification (Duarte et al., 2013; Unsworth et al., 2019a; Ricart et al., 2021). One of the main reasons for the global decline of seagrasses is coastal eutrophication and the subsequent reduction of light availability, which contracts the space where seagrasses can thrive (Cloern, 2001; Orth et al., 2006; Burkholder et al., 2007; Waycott et al., 2009). Light limitation reduces photosynthetic rates of seagrasses, altering their overall carbon balance (Ralph et al., 2007; McMahon et al., 2013), and subsequently leading to net carbon losses which is likely a primary control of seagrass decline (Moreno-Marín et al., 2018; Adams et al., 2020). Indeed, seagrass ecosystems shift to a bare sand state when light availability drops below critical tolerance thresholds, with entire seagrass meadows collapsing after major light limitation events (Walker and McComb, 1992; Preen et al., 1995; van der Heide et al., 2007). For long-term changes in light availability, these thresholds are commonly known as minimum light requirements (MLR). MLR therefore determine the critical light availability for survival of seagrasses over ecologically-relevant time frames and are calculated with light values at maximum colonization depth (Collier et al., 2016). Because of their relevance to sustain meadow persistence, MLR have been identified for numerous seagrass species distributed worldwide (e.g., Erftemeijer and Robin Lewis, 2006; Lee et al., 2007).

Understanding the processes conferring seagrasses with increased resistance to disturbances becomes essential to forecast and prevent the loss of these habitat-forming species. Since light plays the most pivotal role in modulating plant growth and depth limits (Dennison, 1987; Duarte, 1991), changes in seagrass responses to light reduction are a likely pathway by which resistance is conferred to them. Seagrasses respond to light deprivation with a well-defined sequence of changes, the first of which is physiological photoacclimation (Waycott et al., 2005). Physiological photoacclimation refers to the ability of plant leaves to increase their efficiency of converting light into photosynthate and/or decrease respiration demand. The presence or absence of physiological photoacclimation can be identified by measuring changes in both the maximum photosynthetic rates and photochemical efficiency of seagrass leaves in response to changes in their local light environment (Cayabyab and Enríquez, 2007). Under light limitation, plants physiologically acclimate, through two strategies: enhancing light harvesting efficiency (e.g., adjusting metabolic demand of leaf tissues, increasing total chlorophyll, reducing the chlorophyll a:b ratio, etc.), and/or minimizing carbon losses (i.e., carbon allocation strategies) (Olivé et al., 2013; Silva et al., 2013). For instance, the higher content of total chlorophyll (as well as other altered pigments) in the seagrass *Cymodocea nodosa*, together with its carbon allocation strategy, have been argued as the underlying reasons for its superior ability to cope with light deprivation compared to *Zostera marina* (Silva et al., 2013). In addition, seagrasses can also acclimate to low light by increasing shoot size and reducing shoot density (i.e., self-thinning) in order to optimize light capture by the canopy (Enríquez et al., 2019). These strategies are critical for maintaining a positive carbon balance and reducing MLR (Campbell et al., 2007; Silva et al., 2013). However, seagrass species are not all equal and a greater ability to acclimate certain traits can confer plants an improved carbon balance and reduced MLR, allowing seagrasses to survive in environments with lower light availability (Ruiz and Romero, 2001; Minguito-Frutos et al., 2023). Hence the MLR for different seagrass species may depend substantially on the magnitude of its photoacclimation capacity, which itself can be mediated by the contextual conditions where that population resides (Erftemeijer and Robin Lewis, 2006; Cayabyab and Enríquez, 2007). Local contexts, referring to light but also to thermal natural histories, have influenced the strategies of marine macrophytes in responding to abiotic impacts such as light reduction (Ruiz and Romero, 2003; Robledo and Freile-Pelegrín, 2005; Yaakub et al., 2014) or marine heatwaves (Nguyen et al., 2020; Schubert et al., 2021). In particular, seagrasses growing in suboptimal light environments have their MLR altered and show different photosynthetic performance compared to those growing in optimal conditions (Ruiz and Romero, 2003; Yaakub et al., 2014).

**Figure 1:**
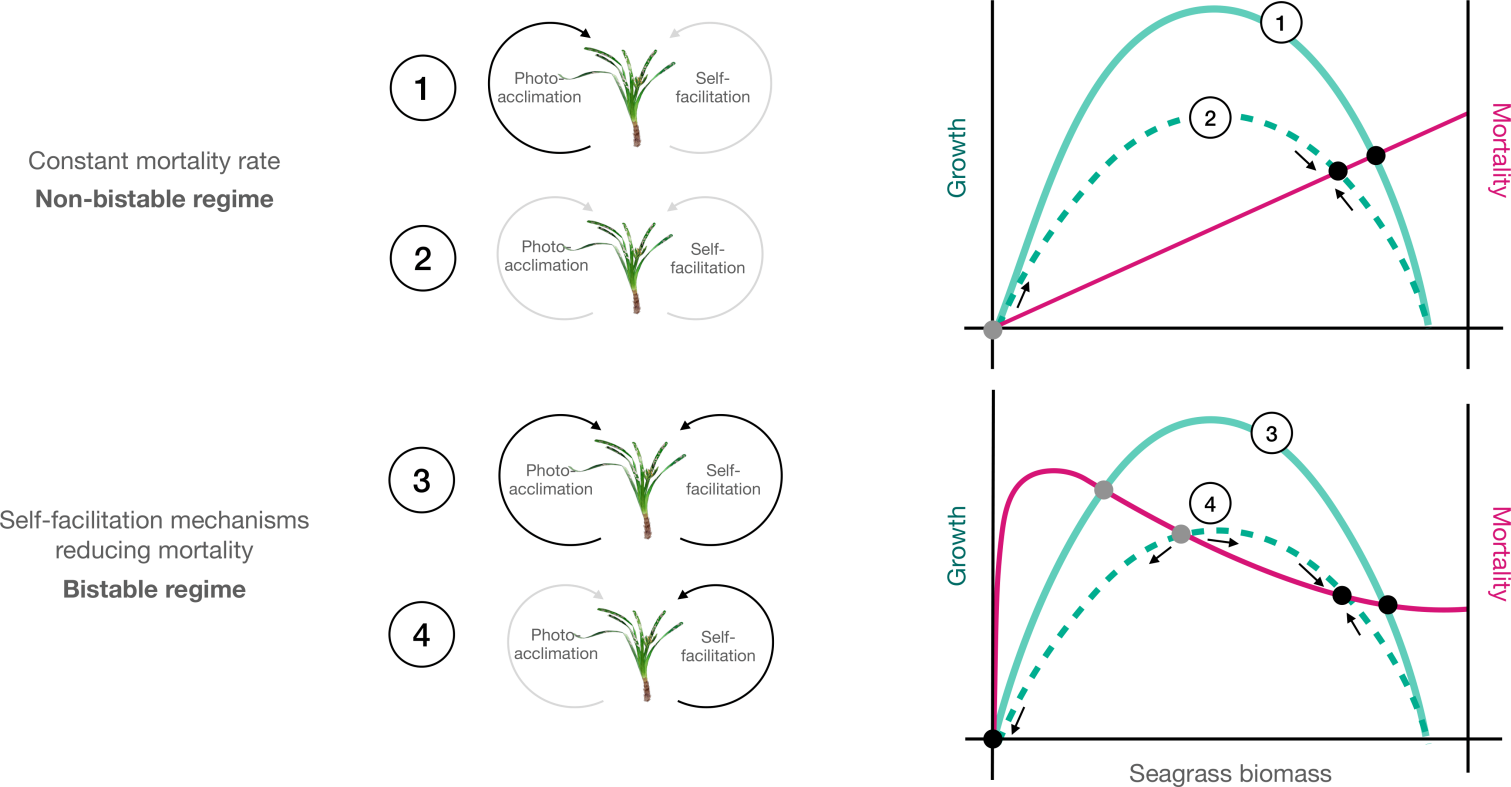
Conceptual diagram describing the different models explored. Green curves represent seagrass growth rates as a function of biomass up to a certain maximum carrying capacity. Red lines represent seagrass mortality rates. Equilibrium points are represented by grey (unstable) and black (stable) dots at the intersection between growth and mortality curves. In the absence of self-facilitation (1 and 2 upper panel), mortality increases with biomass and intersects growth curves forming stable equilibria. In contrast, self-facilitation mechanisms (3 and 4 lower panel) reduce seagrass mortality as biomass increases, promoting the emergence of unstable equilibria leading to bistability (two stable equilibria separated by an unstable equilibrium point). Moreover, plants able to photoacclimate (solid lines) both in non-bistable (1) and in bistable regimes (3) are expected to use light more efficiently and increase their growth, compared to those that cannot (dashed lines 2 and 4).

In addition to these well-defined acclimation responses (Waycott et al., 2005), seagrass ecosystems may also be characterized by the occurrence of feedbacks leading to the emergence of nonlinear dynamics (van der Heide et al., 2007; McGlathery et al., 2013). When sufficiently strong, feedbacks can push seagrass meadows to express bistable behaviors with two possible stable states (seagrass and bare sand) for the same level of external stress (Maxwell et al., 2017; Adams et al., 2018). Bistable behaviors may arise as a consequence of increased light stress provoking abrupt transitions at the deep edge of seagrass meadows (Mayol et al., 2022). For example, if reinforcing mechanisms driven by plant presence exists, and if such mechanisms act to reduce the mortality rates of a stressed seagrass bed (i.e., self-facilitation mechanisms), they can lead to bistability as explored in Mayol et al. (2022) (see **Figure 1**). In that recent work, it was found that bistable behaviors could potentially arise due to clonal integration, which refers to the ability of seagrass species to translocate resources between connected ramets (Terrados et al., 1997; Nielsen and Pedersen, 2000). However, under light stress gradients, mortality increases resulting in shoot density decrease and this lower biomass could significantly modify the local environment by reducing sediment trapping, increasing damage from erosion, reducing physical integration, reducing anchoring and amplifying toxicity from eutrophication (Duarte and Sand-Jensen, 1990; Olesen and Sand-Jensen, 1994; Vidondo et al., 1997; van der Heide et al., 2007; van der Heide et al., 2008; Collier et al., 2009). Overall, seagrasses will display a set of responses to cope with light stress gradients linked to changes in biomass that eventually affect mortality rates, but it is so far unclear whether photoacclimation itself is a nonlinear process able to cause bistability.

Mechanistic models provide a useful tool to explore and untangle the effects of causal processes on ecosystem behavior (Burd and Dunton, 2001). Such models may help to elucidate the effects of physiological photoacclimation and self-facilitation mechanisms (e.g., mechanisms related to a reduction in mortality associated with large biomasses) on identifying MLR and bistability behaviors in seagrass beds. In addition, models incorporating data-calibrated seagrass responses to light reduction can predict seagrass carbon balance (Adams et al., 2020) and quantify their resilience to light limitation (Adams et al., 2018). Hence, predictions of suitably-designed mechanistic models can indicate how plants respond to light reduction (Burd and Dunton, 2001). If seagrasses represented by such models exhibit nonlinear responses and/or alternative stable states, these predictions are critical to inform management actions that could alleviate the light pressure in time before an abrupt change in seagrass ecosystem state, which may be potentially irreversible.

The aim of this study is thus to assess seagrass biomass responses and resilience to four different conditions: the presence (or absence) of physiological photoacclimation as light reduces, and the presence (or absence) of self-facilitation mechanisms (represented here by clonal integration) as biomass reduces. To achieve the study’s aim, we built and parameterized deterministic models of carbon balance using data for the Mediterranean seagrass *Cymodocea nodosa* gathered in field experiments (Marín-Guirao et al., 2022) and field light gradients (Mayol et al., 2022) where both mechanisms have been described. These models do not aim to incorporate all mechanisms influencing seagrass dynamics; rather, the presented models focus on a few relevant mechanisms with the aim of uncovering gaps in scientific knowledge (Burd and Dunton, 2001 and references there in). Our predictions demonstrate how the MLR and potential bistability of seagrass ecosystems is dependent on each of the four proposed models, and thus yield guidance for what new information should be sought if one or more of these ecosystem properties (MLR and bistability) is of interest for decision-making in environmental management.

## 2 Materials and methods

### 2.1 Study system

*Cymodocea nodosa* (Ucria) Ascherson 1869 is a subtidal seagrass species native and widely distributed throughout the Mediterranean, extending in the East Atlantic coasts including the Canary Islands (Garrido et al., 2013). It is considered a fast-growing, medium-size opportunistic species with remarkable phenotypic plasticity that inhabits a broad range of environments, including those with more fluctuating environmental conditions (Olesen et al., 2002; Olivé et al., 2013; Silva et al., 2013; Peralta et al., 2021). *C. nodosa* forms dense monospecific meadows between the water surface and 40 m depth (Terrados and Ros, 1992; Short et al., 2011), exhibiting often abrupt declines in shoot density at their depth limits compatible with alternative stable states caused by self-facilitation mechanisms (Mayol et al., 2022). This plastic seagrass species is characterized by a strong photoacclimation potential, altering its photosynthetic-irradiance (*P-I*) parameters as light is reduced, to optimize its light use efficiency (Olivé et al., 2013; Silva et al., 2013; Marín-Guirao et al., 2022).

### 2.2 Model description

To quantitatively assess the influence of physiological photoacclimation on seagrass ecosystem properties, we examined four models of plant responses to light reduction (**Figure 1**; **Table 1**). In two of the four models, it was assumed that plants photoacclimate (**Figure 1**; scenarios 1 and 3) to changes in light availability by adjusting their photosynthetic-irradiance (*P-I*) parameters (for physiological mechanisms which potentially cause these parameter adjustments see e.g., Marín-Guirao et al., 2022). In the other two models (**Figure 1**; scenarios 2 and 4) and for comparison, it was assumed that plants cannot photoacclimate.

**Table 1:** Equations used in the physiological photoacclimation models.

Eqn.	Model equations	Which scenarios used in?
(1)	$\frac{dB}{dt}=K\frac{1}{1+[BAR]}\left(\frac{1}{2}P-(R+[RRR][BAR])\right)B\;\left(1-\frac{B}{N}\right)-\delta (B)$	①,②,③,④
(2)	$P=\frac{P_{max}I}{I+I_{k}}$	①,②,③,④
(3)	$P_{max}(I)=P_{gmax}+\frac{P_{gmin}-P_{gmax}}{1+e^{\lambda_{P}(I-y_{cP})}}$	①,③
(4)	$I_{k}(I)=I_{kmax}+\frac{I_{kmin}-I_{kmax}}{1+e^{\lambda_{K}(I-y_{cK})}}\;$	①,③
(5)	$R(I)=R_{max}+\frac{R_{min}-R_{max}}{1+e^{\lambda_{R}(I-y_{cR})}}$	①,③
(6)	$\delta (B)=d_{0}B$	①,②
(7)	$\delta (B)=\left(\frac{1+e^{-\lambda_{B}B_{0}}}{1+e^{\lambda_{B}(B-B_{0})}}\right)d_{0}B$	③,④

In the models without photoacclimation, carbon balance is based on the following three *P-I* parameters: maximum gross photosynthetic rate *P_max_* (in units of mg O_2_ g^-1^ above-ground [ABG] dry weight [DW] h^-1^), saturation irradiance *I_k_* (μmol quanta m^-2^ s^-1^) and above-ground respiration *R* (mg O_2_ g^-1^ ABG DW h^-1^). In the models that include photoacclimation, each of these three parameters *P_max_*, *I_k_* and *R* possess a nonlinear dependence on the benthic irradiance *I* (μmol quanta m^-2^ s^-1^) as detailed later in this section. We parameterized our models in terms of the saturation irradiance parameter *I_k_* instead of the (also commonly used) photosynthetic efficiency *α* due to our recent finding that the species-specific ability of seagrasses to acclimatize the parameter *I_k_* better explains these species’ ability to cope with light reduction and colonize depth ranges (Minguito-Frutos et al., 2023). Below-ground respiration by roots and rhizomes (Fourqurean and Zieman, 1991; Burd and Dunton, 2001) was also included in the carbon balance of all four models. Specifically, it was assumed that the rate of below-ground respiration per unit below-ground biomass was 1/10 of the maximum rate of above-ground respiration per unit above-ground biomass (Staehr and Borum, 2011), analogous to ten-fold differences in turnover rate observed between above-ground and below-ground biomass compartments (Vonk et al., 2015). This assumption was mathematically characterized by the constant parameter [*RRR*] = *R_max_*/10 (where [*RRR*] denotes root/rhizome respiration in units of mg O_2_ g^-1^ below-ground [BG] DW h^-1^). The ratio of below-ground biomass to above-ground biomass, required here, was denoted by [*BAR*]. [*BAR*] can be highly variable in the field but typically its order of magnitude is one (see e.g., Pérez et al., 1994; Collier et al., 2017), so for simplicity its default value was set to one in all four models. However, we also explored the effect of increasing [*BAR*] above one in later simulations.

The difference between each of the two models that included photoacclimation and those that excluded photoacclimation was the absence or presence (**Figure 1**; upper and lower panel, respectively) of self-facilitation (clonal integration) reducing mortality rates as biomass increases (see Mayol et al., 2022). We included self-facilitation in our analysis since it may be crucial in favoring natural or induced recovery of seagrasses (van Katwijk et al., 2016; Moksnes et al., 2018). In the models where this self-facilitation was absent, the seagrass mortality rate (*δ*) was set to a constant equal to *d_0_* (in units of h^-1^) so that the biomass lost due to mortality is always proportional to the current biomass of seagrass. In contrast, when self-facilitation was present, this mortality rate (*δ*) depended nonlinearly on the current total biomass of the seagrass state (mortality is reduced at high biomass), as detailed later, in the next section.

All four models used an ordinary differential equation to track the total seagrass biomass *B* (in units of g total DW m^-2^). Models employed the common logistic growth assumption that biomass can accumulate up to some maximum carrying capacity (g total DW m^-2^). The carrying capacity *N* represents, therefore, the maximum population that a system can sustain, depending on various limited resources such as food or space. In our models, we assumed that growth is maximized at *½ N* and that restricted space diminishes production at higher biomass levels as a result of self-shading (Burd and Dunton, 2001).We assumed equal amounts of carbon fixation to the amounts of O_2_ evolved/fixed during photosynthesis-respiration (Fourqurean and Zieman, 1991; Adams et al., 2017), to convert between carbon exchange rates (in units of mg O_2_ g^-1^ ABG DW h^-1^) and growth rates (in units of h^-1^). We firstly introduced a factor (1/1+[*BAR*]), in units of g ABG DW g^-1^ total DW) to account for the products of photosynthesis and respiration allocated to net growth of below-ground tissues. Secondly, we introduced a conversion factor *K* (g total DW mg^-1^ O_2_) to account for the total biomass produced per net mass of oxygen evolved (for further information see **Supplementary Text S1**, Section S1.3). Combining all of the above considerations, the four investigated models all possess the form


(1) $$\frac{dB}{dt}=K\frac{1}{1+[BAR]}\left(\frac{1}{2}P-(R+[RRR][BAR]\right)B\;\left(1-\frac{B}{N}\right)-\delta (B)$$

where the factor of 1/2 in front of the gross photosynthesis rate term *P* accounts for the average difference in time over which photosynthesis and respiration (*R*) processes are occurring (12 hours per day vs 24 hours per day, respectively). The gross photosynthesis rate *P* (mg O_2_ g^-1^ ABG DW h^-1^) is implemented using the standard Michaelis-Menten formulation (Pérez and Romero, 1992; Marín-Guirao et al., 2022; Mayol et al., 2022),


(2) $$P=\frac{P_{max}I}{I+I_{k}}$$

where *I* is the instantaneous light (photosynthetically active radiation, in units μmol quanta m^-2^ s^-1^).

### 2.3 Modelling physiological photoacclimation to light limitation

In the two models where photoacclimation is absent, the parameters for photosynthesis and respiration (*P*_max_, *I*_k_ and *R*) are set to constant values (corresponding to observed values of these parameters in seagrasses acclimated to high light conditions). Conversely, in the two models where photoacclimation is present, these three parameters are assumed to possess a nonlinear dependence on the benthic irradiance *I*. Here we provide justification for the mathematical formulations *P*_max_(*I*), *I*_k_(*I*) and *R*(*I*) that are assumed in the two photoacclimation models.

**Figure 2:**
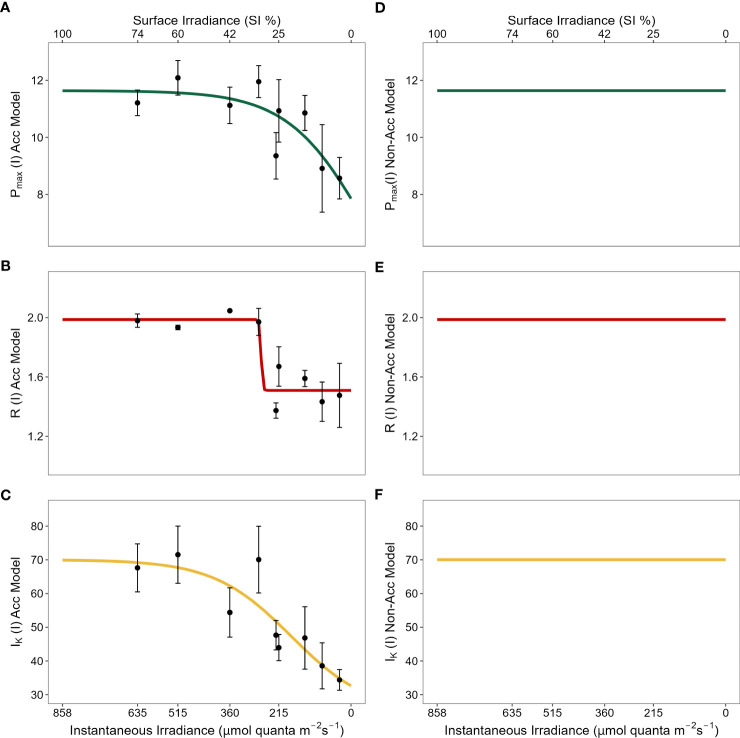
Physiological photoacclimation models compared (left vs right panels) under the light stress gradient. Y-axes represent values for each physiological mechanism along the gradient: maximum gross photosynthesis [*P_max_*, **(A)** vs **(D)**]; above-ground respiration [*R*, **(B)** vs **(E)**]; and saturation irradiance [*I_k_*, **(C)** vs **(F)**]. *P_max_* and *R* are shown in units of mg O_2_ g^-1^ ABG DW h^-1^ and *I_k_* in μmol quanta m^-2^ s^-1^. X-axes represent light values of total instantaneous irradiance (μmol quanta m^-2^ s^-1^) (bottom) and percentage of surface irradiance (SI) (top). X-axes values ranged from 858.04 μmol quanta m^-2^ s^-1^ of instantaneous irradiance that equaled 100% SI after conversions derived from field shading experiments. While lines in left panels represent the best fit curve to the nine levels of light stress (black dots corresponding to 74, 60, 42, 32, 26, 25, 16, 10 and 4% SI) for each physiological mechanism in field shading experiments; lines in right panels are set to constant values corresponding to their maximum values observed at high light conditions.


Based on the data shown in **Figure 2** (adapted from Marín-Guirao et al., 2022; see also **Supplementary Table S1**), all three parameter functions *P*_max_(*I*), *I*_k_(*I*) and *R*(*I*) can be feasibly represented by sigmoidal functions (curves in **Figure 2**; **Supplementary Table S2**). Thus, for the photoacclimation models we assumed that:

(3) $$P_{max}(I)=P_{gmax}+\frac{P_{gmin}-P_{gmax}}{1+e^{\lambda_{P}(I-y_{cP})}}$$

(4) $$I_{k}(I)=I_{kmax}+\frac{I_{kmin}-I_{kmax}}{1+e^{\lambda_{K}(I-y_{cK})}}$$

(5) $$R(I)=R_{max}+\frac{R_{min}-R_{max}}{1+e^{\lambda_{R}(I-y_{cR})}}$$

In equations (3) and (4) that are related to photosynthesis, *P_gmax_* and *I_kmax_* represent the maximum gross photosynthetic rate for seagrass acclimated to high light conditions (mg O_2_ g^-1^ ABG DW h^-1^) and the saturation irradiance for seagrass acclimated to high light conditions (μmol quanta m^-2^ s^-1^), respectively. As irradiance *I* declines, the values of these two parameters decline towards minimum values: *P_gmin_* which represents the maximum gross photosynthetic rate for seagrass acclimated to low light conditions, and *I_kmin_* which represents the saturation irradiance for seagrass acclimated to low light conditions. The slopes (*λ_P,_ λ_K_*) and the inflection points (*y_cP_*, *y_cK_*) of the curves in equations (3) and (4) determine the strength of the declines for *P*_max_ and *I_k_*, respectively (**Table 2**). Similarly, in the above-ground respiration equation (5), *R_max_* and *R_min_* represent the leaf respiration rates (mg O_2_ g^-1^ ABG DW h^-1^) for seagrass acclimated to high and low light conditions respectively, and the slope *λ_R_* and the inflection point *y_cR_* have analogous definitions to *λ_P,_ λ_K_* and *y_cP_*, *y_cK_* (**Table 2**).

**Table 2:** Values of models parameters.

Parameter	Value	Description	Units	Reference
Non-acclimation Model				
*P_gmax_*	11.64	Maximum Photosynthesis	*mg O*_2_/(*g ABG DW* · *h*)	Marín-Guirao et al., 2022
*I_kmax_*	70.04809	Saturation Irradiance	*µmol quanta m* ^-2^ *s* ^-1^	Marín-Guirao et al., 2022
*R_max_*	1.987	Above-ground Respiration	*mg O*_2_/(*g ABG DW* · *h*)	Marín-Guirao et al., 2022
Acclimation Model				
*P_gmax_*	11.64	*P_max_* (high light)	*mg O*_2_/(*g ABG DW* · *h*)	Marín-Guirao et al., 2022
*P_gmin_*	2.096	*P_max_* (low light)	*mg O*_2_/(*g ABG DW* · *h*)	Marín-Guirao et al., 2022
*λ_P_*	0.008524	*P_max_* Slope	*µmol quanta m* ^-2^ *s* ^-1^	
*y_cP_*	-49.1	*P_max_* Threshold	*µmol quanta m* ^-2^ *s* ^-1^	
*I_kmax_*	70.04809	*I_k_* (high light)	*µmol quanta m* ^-2^ *s* ^-1^	Marín-Guirao et al., 2022
*I_kmin_*	24.54396	*I_k_* (low light)	*µmol quanta m* ^-2^ *s* ^-1^	Marín-Guirao et al., 2022
*λ_K_*	0.00863	*I_k_* Slope	*µmol quanta m* ^-2^ *s* ^-1^	
*y_cK_*	177.45783	*I_k_* Threshold	*µmol quanta m* ^-2^ *s* ^-1^	
*R_max_*	1.987	Above-ground Respiration (high light)	*mg O*_2_/(*g ABG DW* · *h*)	Marín-Guirao et al., 2022
*R_min_*	1.509	ABG Respiration (low light)	*mg O*_2_/(*g ABG DW* · *h*)	Marín-Guirao et al., 2022
*λ_R_*	0.4567	ABG Respiration Slope	*µmol quanta m* ^-2^ *s* ^-1^	
*y_cR_*	267.1	ABG Respiration Threshold	*µmol quanta m* ^-2^ *s* ^-1^	
Below-ground Parameters				
[*BAR*]	1	Below-Above Ratio	–	Pérez et al., 1994; Collier et al., 2017
[*RRR*]	0.1987	Roots/Rhizomes Respiration	*mg O*_2_/(*g BG DW* · *h*)	Staehr and Borum, 2011
Biomass and Growth/Mortality Parameters				
*K*	0.001075	Conversion Efficiency	*g total DW/mg O*_2_	Nielsen and Pedersen, 2000; Zharova et al., 2008
*N*	100	Carrying Capacity	*g ABG DW m* ^-2^	
*d_0_*	0.000116	Mortality Rate	h^-1^	Mascaró et al., 2014
*λ_B_*	0.05	Facilitation Slope	(*g ABG DW m* ^-2^)^-1^	Mayol et al., 2022
*B_0_*	10	Threshold Facilitation	*g ABG DW m* ^-2^	Mayol et al., 2022

Full justification of these parameter values is provided in **Supplementary Text S1**.

These curves were fitted to the data obtained in field experiments (Marín-Guirao et al., 2022) by employing a robust and efficient implementation of the Levenberg-Marquardt algorithm for solving nonlinear least squares problems (via the *minpack.lm* R package; Elzhov et al., 2022). The nonlinear least-squares estimates of the parameters obtained from this model-data fitting, along with other parameters sourced from the literature for use in our models, are listed in **Table 2** and justified in **Supplementary Text S1**.

### 2.4 Modelling seagrass mortality

To test the influence of seagrass mortality in the carbon balance models we considered two different mortality functions. In the first function, we assume the absence of self-facilitation (**Figure 1**; scenario 1 and 2), so seagrass mortality (*δ*) responds constantly (*d*_0_) to the current seagrass biomass (*B*) and presents the form:


(6) $$\delta (B)=d_{0}B$$

In the second function, self-facilitation (**Figure 1**; scenario 3 and 4) is assumed to yield a nonlinear relationship between the mortality rate (*δ*) and seagrass biomass (*B*) according to (Mayol et al., 2022),


(7) $$\delta (B)=\left(\frac{1+e^{-\lambda_{B}B_{0}}}{1+e^{\lambda_{B}(B-B_{0})}}\right)\;d_{0}B$$

where parameters *λ_B_* (units of inverse biomass) and *B*_0_ (units of biomass) control the shape of the nonlinear relationship between mortality rate and seagrass biomass.

### 2.5 Solving the models: determining minimum light requirements

To quantify how the four presented models of seagrass carbon balance are affected by the reduction of benthic irradiance *I*, we performed numerous mathematical evaluations of the model expression for dB/dt shown in Eqn. 1. To do so, we assumed that daily averaged light *I_daily_* (i.e., light dose over one day) was approximately equal to half of a constant instantaneous light value *I* received during daylight hours, i.e., *I_daily_* (mol quanta m^-2^ d^-1^) in Marín-Guirao et al. (2022) ≈ *½ I* (instantaneous irradiance in μmol quanta m^-2^ s^-1^). We then calculated the equivalent percentage of surface irradiance (SI = 100%-0%), and solved all models up to their carrying capacity (*N* = 100 g total DW m^-2^). After solving the four models of seagrass response to limiting light conditions, we determined the MLR of each model as the minimum quantity of benthic irradiance (both as instantaneous light received during daylight hours in μmol quanta m^-2^ s^-1^ and as daily averaged light in % SI) that allows a positive rate of change for seagrass biomass, dB/dt (g total DW m^-2^ h^-1^).

To evaluate the influence of physiological photoacclimation and self-facilitation (clonal integration) on the stability of seagrass ecosystems, we calculated the equilibrium points resulting from each of the models. These equilibrium points can be stable (**Figure 1** *black points*) or unstable (**Figure 1** *gray points*), depending on whether the system tends towards an equilibrium point or moves away from them, respectively. When self-facilitation is present, the system can potentially express two stable equilibria separated by an unstable equilibrium point (a situation known as bistability). However, when self-facilitation does not occur, unstable equilibrium points are not expected to emerge and bistability would not take place. Thus, we used the R *rootSolve* package (Soetaert, 2009), which identifies all the equilibrium points within the pre-specified range, as well as whether they represent stable or unstable equilibria.

### 2.6 Quantifying the ecological resilience to light limitation

Ecological resilience was quantified following the definition in Adams et al. (2018) for those models that expressed bistability:


(8) $$Resilience=\frac{B_{stable}-B_{unstable}}{B_{stable}}\times 100\%$$

In this equation, *B*_stable_ and *B*_unstable_ are the strictly positive values of seagrass biomass (g DW m^-2^) representing stable and unstable equilibrium points, respectively. In bistable ecosystems, the ecological resilience calculated from Eqn. (8) will always fall between 0% and 100%, with the system being more resistant to disturbance as the calculated resilience approaches 100%.

## 3 Results

**Figure 3:**
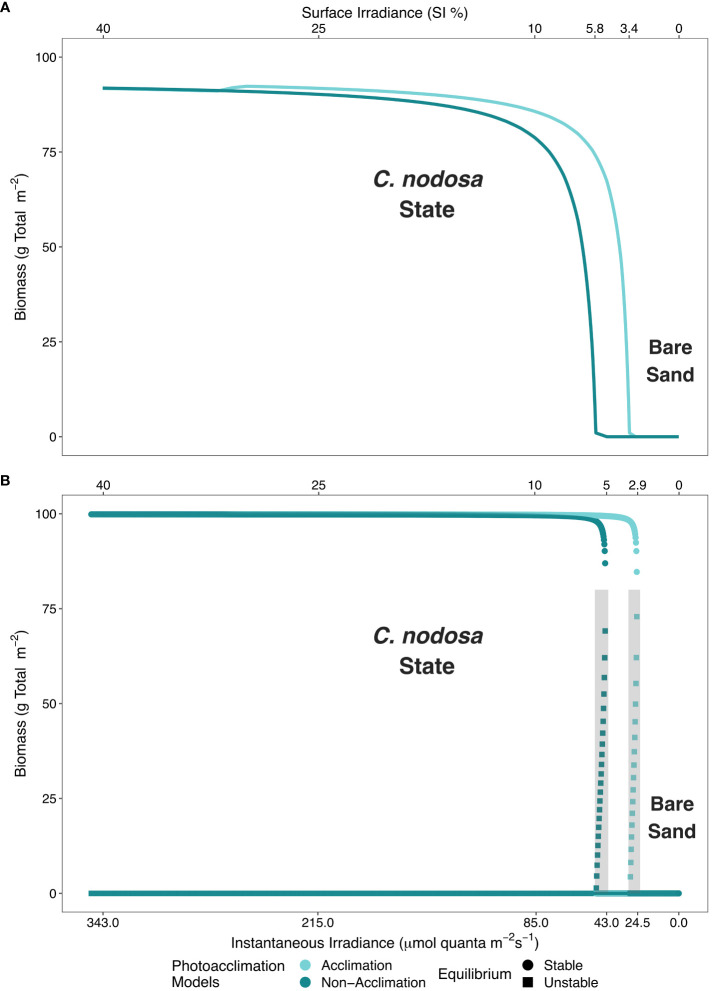
Minimum light requirements (MLR) are depicted for the four presented models: **(A)** physiological photoacclimation and non-acclimation models in absence of self-facilitation (clonal integration) and, **(B)** photoacclimation models in presence of self-facilitation. Physiological photoacclimation models are shown in light blue (acclimation) and dark blue (non-acclimation). In the presence of self-facilitation, bistability arises and circles (stable) and squares (unstable) represent equilibrium types. The shaded area in **(B)** represents the bistable region predicted by the models.

Our models predicted that photoacclimation decreases the MLR of *Cymodocea nodosa* from 5.8% SI to 3.4% SI (**Figure 3A**), when other nonlinear mechanisms (i.e., reduction in mortality rate from clonal integration) are absent. Photoacclimation does not result in bistable behaviors of seagrass ecosystems (one stable equilibrium for each model in **Figure 3A**), despite it being a nonlinear process itself.

The inclusion in our models of the clonal integration mechanism (i.e., self-facilitation), also reduced the MLR, but not as substantially as the photoacclimation mechanism. Models predicted MLR of *C. nodosa* to be 5.1% SI and 2.9% SI in the absence and presence of photoacclimation, respectively (**Figure 3B**), when self-facilitation was introduced.

**Figure 4:**
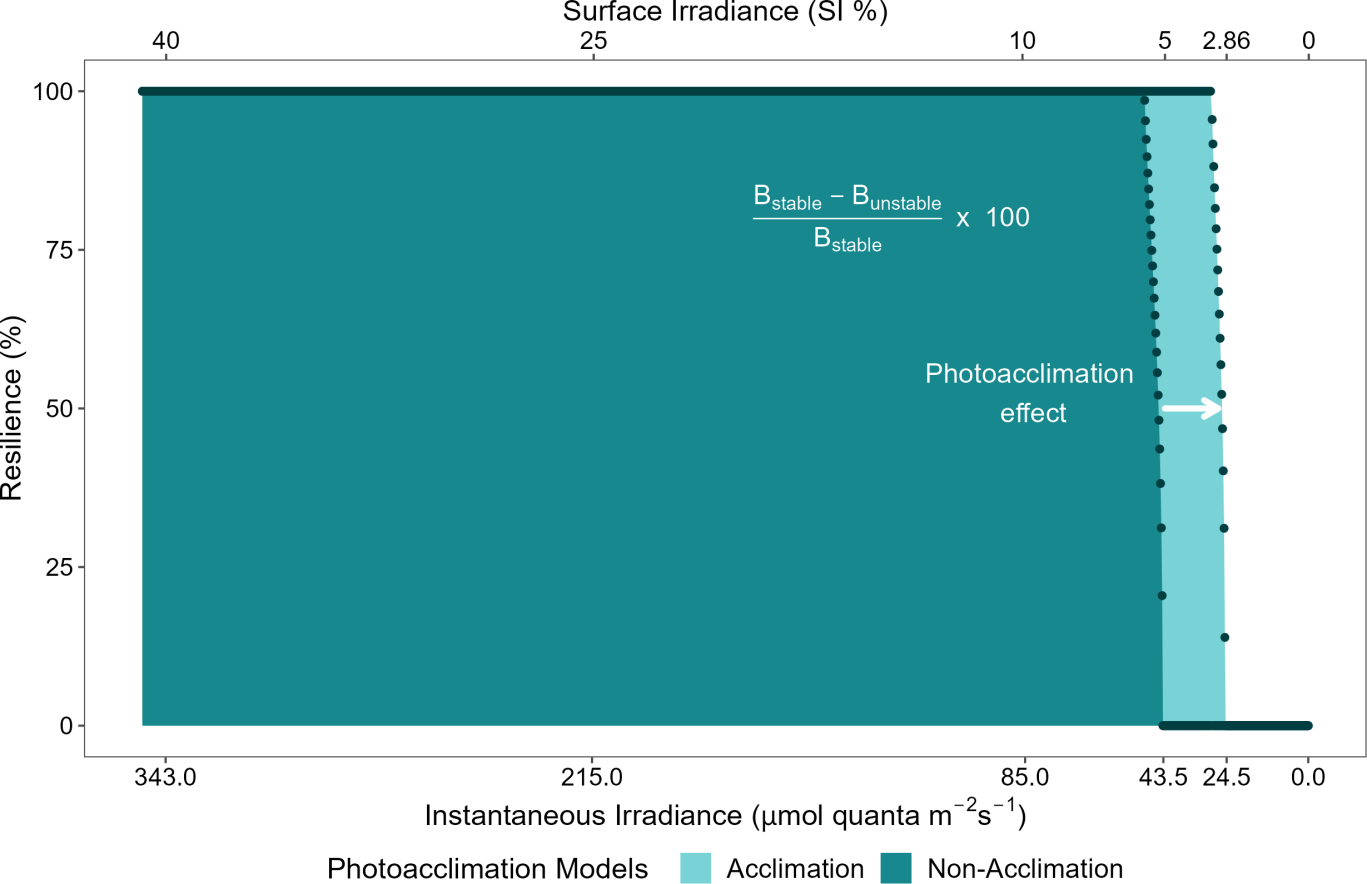
Ecological resilience for physiological photoacclimation (light blue) and non-acclimation (dark blue) models to light limitation, as a function of benthic irradiance (in instantaneous irradiance and % SI). Resilience is calculated following the mathematical definition provided in Adams et al. (2018) which has its basis in the conceptual definition provided in Gunderson (2000). Dots represent the calculated values of resilience (%) as a function of the values of instantaneous irradiance (µmol quanta m^-2^ s^-1^) or surface irradiance (%SI), for each model.

The self-facilitation (i.e., mortality reduction as plant biomass increases) also yielded bistability within specific ranges of benthic irradiance values, regardless of whether photoacclimation was present or not (shaded areas in **Figure 3B**). Hence, when photoacclimation was absent, the seagrass ecosystem formed by *C. nodosa* was bistable when the average daily irradiance was between 5.1% SI and 5.8% SI; and when photoacclimation was present, this ecosystem was bistable when the average daily irradiance was between 2.9% SI and 3.4% SI. The photoacclimation mechanism also permitted a greater range of irradiance values over which the seagrass has maximal resistance to disturbance (**Figure 4**).

**Figure 5:**
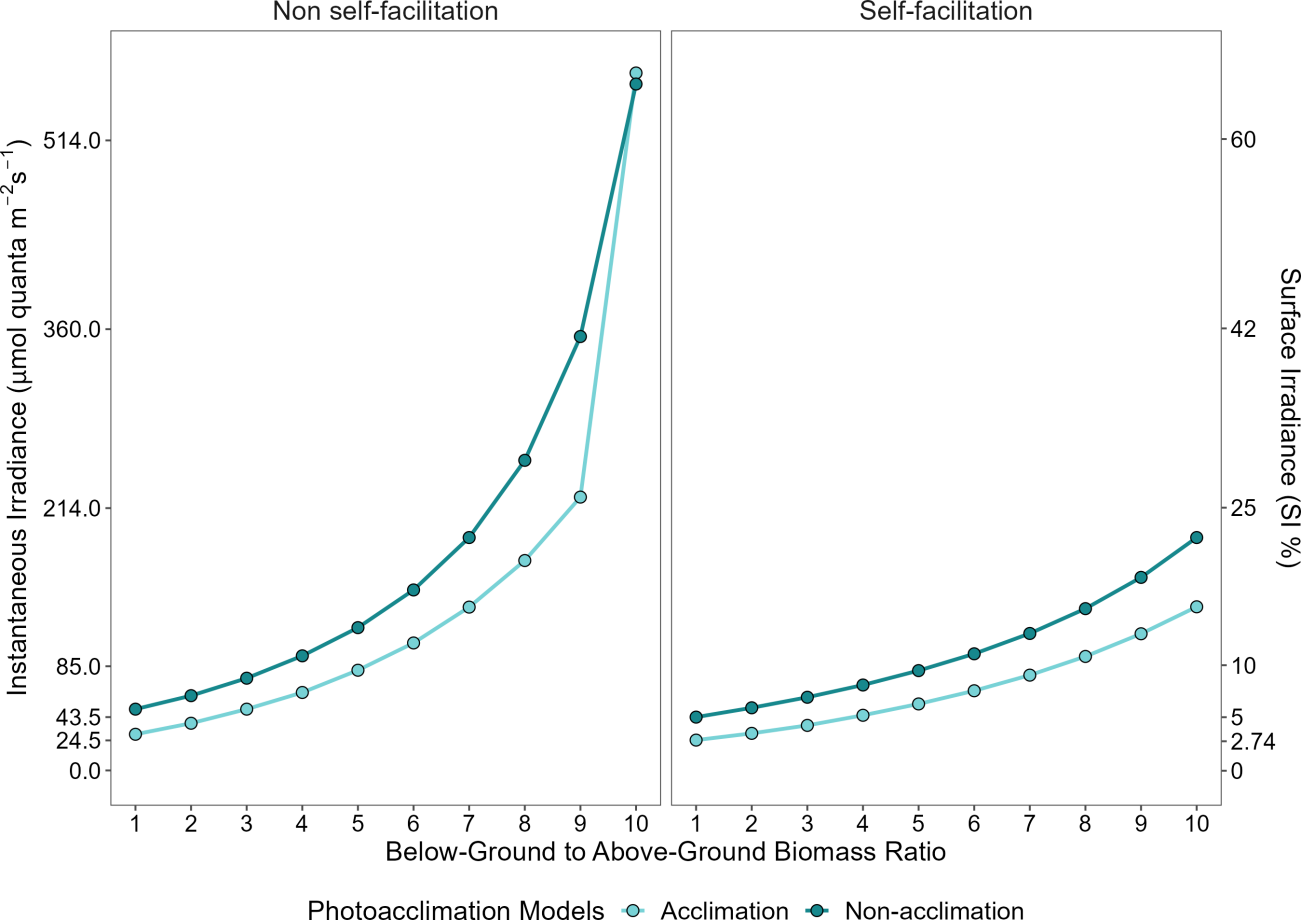
Predicted minimum light requirements as a function of below-ground to above-ground biomass ratio (model parameter [*BAR*]), for plants that express physiological photoacclimation (light blue) or not (dark blue). Left and right panels represent the absence and presence of self-facilitation, respectively.

We also explored the effect of changing the below-ground to above-ground biomass ratio on the MLR predicted by our four models. Crucially, we found that as the ratio of below-ground biomass to above-ground biomass ([*BAR*]) increases, there is a substantial increase in the MLR for plants both with and without photoacclimation capacities (**Figure 5**). However, plants that photoacclimate cope better with a relative increase of the biomass ratio between below- and above-ground tissues, except when self-facilitation is absent and a threshold is exceeded (MLR at [*BAR*] equal to 9, ~26.2% SI compared to ~41.5% SI) (**Figure 5** left panel). After this threshold ([*BAR*] = 9) the MLR of *C. nodosa* that do not possess self-facilitation mechanism increases abruptly. On the contrary, in a system where self-facilitation is acting, and [*BAR*] is equal to 10, the MLR of *C. nodosa* increases gradually – together with photoacclimation the MLR only reaches ~15.6% SI (**Figure 5** right panel).

## 4 Discussion

In this modelling study, we used data from intensive field experimentation to test the effect of physiological photoacclimation on the minimum light requirements (MLR) and, then resilience, of seagrasses performing contrasting physiological strategies to cope with light stress gradients. Specifically, our models predict that the ability of *C. nodosa* to photoacclimate to low light approximately halves its threshold of collapse (MLR). In addition, the presence of self-facilitation mechanisms such as clonal integration, alleviating mortality rates with increasing biomass, can result in bistability and decrease MLR even further. On the other hand, photoacclimation increases the resilience of bistable meadows to light reduction by increasing plant resistance and their ability to recover (O’Brien et al., 2018), but it cannot yield bistability in seagrass beds. Therefore, the results from our models point to the critical role of physiological photoacclimation in conferring resilience to seagrasses against light deprivation, and also illustrate that other non-linear mechanisms (if present) can cumulatively contribute to this resilience and shape the recovery of seagrass ecosystems exposed to light reduction.

Using our modelling approach, we provide a coarse quantification of the MLR of *C. nodosa*, which can reorganize its photosynthetic apparatus to resist light limitation, and compare this to the equivalent MLR predicted if such plants are unable to photoacclimate. Our study uses data from field shading experiments that identified patterns of physiological acclimation mechanisms of *C. nodosa* to light-limiting conditions (see Marín-Guirao et al., 2022). MLR predicted through our mathematical modelling approach (2.9% – 3.4% SI for plants with and without self-facilitation respectively) are comparable to those found by previous experimental studies (e.g., 4.4% SI (Olesen et al., 2002) and 7.3% – 10.2% SI (Dennison et al., 1993)). Of particular note, a halving of MLR can yield a contraction of ≈1 m to ≈10 m at the deep limit, depending on the values of the light attenuation coefficient (*k =* 0.07 to *k* = 0.57 respectively, see Dennison et al., 1993; as calculated using the standard light extinction equation provided in Dennison, 1987).

The photoacclimation-mediated reduction in MLR is mainly related to *C. nodosa*’s capacity to decrease both its leaf respiration demand and saturation irradiance *I_k_* as benthic irradiance decreases. Reducing leaf respiratory losses is critical for seagrasses to survive periods of light stress, as leaf respiration typically accounts for the majority of respiratory requirements in seagrasses (Fourqurean and Zieman, 1991; Masini et al., 1995). The decline in above-ground respiration observed at low light environments is a common photoacclimation mechanism in *C. nodosa* (Olivé et al., 2013; Marín-Guirao et al., 2022), but also among other seagrass species, making the outcomes found in this study potentially applicable to other seagrasses (Ruiz and Romero, 2001; Ruiz and Romero, 2003; Collier et al., 2009; Collier et al., 2012). In fact, several species of different genera have been found to reduce their above-ground tissues to balance the energetic demand under impoverished light environments (Mackey et al., 2007; Collier et al., 2012). Similarly, some seagrass species have been shown to reduce their total leaf area (i.e., self-thinning) as a strategy to cope with light reduction at depth, increasing their overall production (Enríquez et al., 2019). Our finding that reduced *I_k_* at low light levels boosts the carbon balance of *C. nodosa* agrees with previous findings for this species (Olivé et al., 2013) and other seagrasses (Ruiz and Romero, 2001; Campbell et al., 2007). Interestingly, we also found that even though *P_max_* decreases as benthic irradiance declines, this did not cause a detrimental effect on carbon production compared to plants unable to photoacclimate (constant *P_max_*). This occurred because the reductions in both respiration demand and *I_k_* of plants able to acclimate, counteract the simultaneous reduction of *P_max_*, as light decreases. Therefore, in the present work we have been able to demonstrate how the cumulative changes in multiple physiological parameters at low light, allow seagrasses to more efficiently harvest light, compared to those plants with less variability in their physiological response. These results are in agreement with field experiments that found the plastic *C. nodosa* to be more resilient under adverse light conditions compared to other less plastic seagrasses like *Zostera marina* (Silva et al., 2013) or *Posidonia oceanica* (Olesen et al., 2002).

The results of our work show that presence of self-facilitation mechanisms, such as clonal integration leading to reduced mortality rates with increasing biomass, further reduces the MLR of *C. nodosa*, but also makes the ecosystem prone to bistability. Clonal integration might be the main reinforcing feedback in clonal seagrass plants (Nielsen and Pedersen, 2000), although a wide range of reinforcing feedbacks (e.g., enhancing sediment trapping, providing physical protection, etc.) could act similarly in reducing seagrass mortality with increasing biomass (Burkholder et al., 2007; Maxwell et al., 2017). Unlike self-facilitation mechanisms, photoacclimation alone did not cause bistability in the ecosystem formed by *C. nodosa*. Thus, we show that nonlinear trajectories in acclimation mechanisms (i.e., photoacclimation) do not always lead to the emergence of bistability and may not always be the cause of alternative stable states (McGlathery et al., 2013), but are still drivers of threshold behaviors that challenge the management of these ecosystems (van Katwijk et al., 2016). The presence of multiple alternative states (i.e., seagrass and bare sand states) for a given level of irradiance (i.e., bistability) is an indicator of hysteresis, which carries significant additional impediments for the recovery of the ecosystem as multiple feedbacks acting in the alternative state can reinforce its persistence (Moksnes et al., 2018). Given the prevalence of self-facilitation mechanisms alleviating mortality, the presence of bistability is a plausible scenario (van der Heide et al., 2007; Carr et al., 2010) in the ecosystems sustained by seagrasses independently of their photoacclimation capacities.

As is the case for many coastal ecosystems, seagrasses are exposed to multiple coexisting stressors (hurricanes or storms, overgrazing, etc.) eroding their resilience (Ruiz et al., 2009; Carlson et al., 2010; Infantes et al., 2011). These disturbances often cause uneven distributions of below- and above-ground plant tissues (i.e., leaves and rhizomes), eventually altering the below-ground to above-ground biomass ratio ([*BAR*]). We found that this ratio is essential for understanding seagrasses resilience to light-limiting conditions. As [*BAR*] increases, MLR increase too, making *C. nodosa* more vulnerable to light limitation. High below-ground to above-ground biomass ratios (e.g., [*BAR*] ≥ 10) can be particularly challenging for maintaining positive carbon balance after heavier losses of photosynthetic tissues due to herbivory (Ruiz et al., 2009). In these situations, the effect of the physiological photoacclimation on increasing seagrass resilience to light deprivation may not be sufficient to counteract the effects of external stressors unless other compensatory mechanisms are also present (e.g., reduced respiration in the below-ground tissues, not investigated in this study). Reallocation of plant material between below-ground and above-ground tissues could also potentially be a strategy to boost carbon availability in periods when photosynthesis is suboptimal (Zimmerman et al., 1995; Alcoverro et al., 1999). However, large below-ground structures can also paradoxically be considered a heavy burden in periods of reduced light due to their respiratory requirements (Fourqurean and Zieman, 1991; Hemminga, 1998; Alcoverro et al., 2001). For this reason, further investigation is required to understand the actual role of below-ground tissues, which may increase the predictive power of seagrass production models and, therefore, their utility for making effective management decisions (Burd and Dunton, 2001). In the current era of global change, understanding the limits of acclimation capacity under the cumulative effects of human impacts on seagrasses influencing their resilience becomes imperative (Adams et al., 2020).

Overall, the goal of these models was to assess the effect of *C. nodosa*’s photoacclimation capacity on reducing their MLR (increased resistance) and enhancing its resilience to light deprivation. To accomplish this, our models included few essential mechanisms, making easier to understand the gaps in our knowledge and did not include all relevant processes influencing seagrass carbon balance (Burd and Dunton, 2001). Given this, we found that in all the scenarios explored, transitions followed non-linear dynamics. However, unlike self-facilitation, photoacclimation did not lead to bistability of *C. nodosa*’s seagrass beds. Nonlinearities carry critical implications for the predictability of abrupt shifts in the ecosystems formed by seagrasses, particularly for those exhibiting bistable behaviors (due to potential hysteresis). Our results foresee that under increasing light disturbances related to global change (Unsworth et al., 2019b), seagrass species that have evolved greater photosynthetic plasticity might be less vulnerable to anthropogenic reductions in light availability. Equally important, our models demonstrate that seagrass ecosystems that possess self-reinforcing mechanisms to reduce their mortality rates when biomass is high can be bistable.

Since not all species are equally able to acclimate to light reduction (Minguito-Frutos et al., 2023) nor do the meadows they form possess the same ecosystem components, there is an imminent need to identify differences in species acclimation capacity and prevalence of feedback mechanisms (Maxwell et al., 2017). This will help create management plans that account for species-specific vulnerability to stressors as light deprivation. Considering that light limitation is the main factor affecting seagrasses at different organizational scales: physiological, morphological and population (Ralph et al., 2007), our study therefore contributes to an improved understanding of seagrass ecosystem functioning which is valuable for conservation efforts. Specifically, we show how plastic seagrasses such as *C. nodosa* with potentially multiple reinforcing feedbacks give a wider operational space for managerial action to conserve the ecosystems formed by these species. Continued strategies to manage and conserve seagrass ecosystems can help to avoid sudden collapses in seagrass beds, which in some cases could be potentially irreversible (Lee et al., 2007; van der Heide et al., 2007).

## Data availability statement

The original contributions presented in the study are included in the article/**Supplementary Material**, further inquiries can be directed to the corresponding author/s.

## Author contributions

All authors contributed to the study design. MM-F, MA, DA, EM, TA, and JB led the modelling and statistical analyses. LM-G, JB-E and JR carried out the *Cymodocea nodosa* field experiment that served as the basis of this modeling study. MM-F, MA, MV, TA, and JB led the writing of the manuscript with contributions of all the authors. All authors contributed to the article and approved the submitted version.
