# Supplementary material for: Quantifying the role of photoacclimation and self-facilitation for seagrass resilience to light deprivation
Source: Front Plant Sci. 2023 Jul 21;14:1186538. doi: 10.3389/fpls.2023.1186538 (PMC10401047; doi:10.3389/fpls.2023.1186538)
Supplement: Supplementary file 1 [file Table_1.docx]

Supplementary Material

Quantifying the role of photoacclimation and self-facilitation for seagrass resilience to light deprivation

Mario Minguito-Frutos^1*^, Matthew P. Adams, Teresa Alcoverro, María P. Vilas, David Alonso, Elvira Mayol, Jaime Bernardeu-Esteller, Lázaro Marín-Guirao, Juan M. Ruiz, Jordi Boada

*** Correspondence:** Mario Minguito-Frutos: [mminguito@ceab.csic.es](mailto:mminguito@ceab.csic.es)

# Supplementary Text S1: Justification of parameter values

The full list of model parameters used in this paper and their references are shown in Table 1. Here, we provide a deeper justification of the parameter values, from those obtained through field experiments to those adapted from literature.

**S1.1 Photosynthetic – Irradiance (*P-I*) parameters against light reduction**

Marín-Guirao et al. (2022) found, after two months of experimental shading in the field, the pattern of physiological photoacclimation represented by dots in Fig. 2. Authors measured the maximum gross photosynthetic rates (*P_max_*, in units of μmol 0_2_ g^-1^ ABG FW h^-1^); above-ground respiration (*R*, in units of μmol 0_2_ ABG g^-1^ FW h^-1^); saturation irradiance (*I_k_*, in units of μmol quanta m^-2^ s^-1^) and compensation irradiance (*I_c_*, in units of μmol quanta m^-2^ s^-1^). To do so, they tested 9 values of light reduction with respect to the natural incoming light: 0%, 18%, 43%, 56%, 64%, 66%, 78%, 87% and 94% of subsurface irradiance (SI). That experiment yielded a control value (no light reduction) of 74% SI, which after conversions to instantaneous irradiance resulted in 634.96 μmol quanta m^-2^ s^-1^. This implies a maximum value of instantaneous irradiance (100% SI) that is equivalent to 858.04 μmol quanta m^-2^ s^-1^. Thus, the shade screens produced an 8-level light reduction gradient corresponding to 60, 42, 32, 26, 25, 16, 10, 4% SI, which are equivalent to 514.82, 360.42, 274.54, 223.14, 214.58, 137.26, 85.88, 34.26 μmol quanta m^-2^ s^-1^, respectively.

In this work, we created two models of carbon balance, to quantitatively evaluate the contribution of physiological photoacclimation on seagrass response when facing such levels of light reduction. One of these models assumed the pattern obtained in Marín-Guirao et al. (2022) for *Cymodocea nodosa.* In the other and for comparison, it was assumed that plants cannot photoacclimate. To do this so, values of *P_max_* and *R* found in Marín-Guirao et al. (2022) needed to be converted from units of μmol 0_2_ g^-1^ ABG FW h^-1^ to units of mg 0_2_ ABG DW h^-1^. Therefore, we made the conversions from mol to mg with 32 g 0_2_ corresponding to 1 mol 0_2_ (Pérez and Romero, 1992) and from FW to DW with 3.37 g FW corresponding to 1 g DW (Marco-Méndez, 2015). *I_k_* and *I_c_* maintained the same units (μmol quanta m^-2^ s^-1^).

Once the values were transformed (Table S1), we applied a nonlinear fitting procedure to determine the nonlinear least-squares estimates of the model parameters presented in equations (3)-(5) in the manuscript text. We used a robust and efficient implementation of the Levenberg-Marquardt algorithm (via the R package 'minpack.lm'). The estimated values of model parameters estimated from this fitting procedure were used in subsequent model simulations; the full list of these model parameters is shown in Table S2.

**S1.2 Below-ground parameters**

To set the value of below-ground respiration (roots-rhizomes respiration [*RRR*]) we used the ratio obtained for *Zostera marina* by Staehr and Borum (2011) as follows. They found that root and rhizome respiration rates were nearly 10-fold lower than above-ground respiration rates (leaves), analogous to ten-fold differences in turnover rate observed between above-ground and below-ground biomass compartments (Vonk et al., 2015). This respiration ratio between photosynthetic and non-photosynthetic tissues was independent of seasonal and temperature changes (Staehr and Borum, 2011). Therefore, we set a value of the *RRR* parameter to be equal to 1/10 of *R_max_* (maximum leaf respiration).

The ratio [*BAR*] between below-ground biomass and above-ground biomass was set to one, following the findings on Pérez et al. (1994). This ratio is ~1 for high and medium nutrient-mediated seagrass beds of *C. nodosa*. While this ratio is largely dependent on the seasonality and local context of seagrass beds, a below-ground to above-ground biomass ratio of ~1 has also been found for some seagrass beds of its congener species *Cymodocea serrulata* (Collier et al., 2017 and references therein).

**S1.3 Biomass and growth parameters**

Given that our carbon balance model was written in units of mg 0_2_ g^-1^ ABG DW h^-1^, we had to include a conversion factor (K; in units of g total DW mg^-1^ 0_2_) to obtain the growth rates (in units of h^-1^) for *Cymodocea nodosa*. To parameterize the value of K, we considered the maximum values of above-ground growth rates (μ_max_) known for *C. nodosa* (Zharova et al., 2008; Nielsen and Pedersen, 2000) and solved our model for maximum net carbon production of *C. nodosa*:

$$\mu_{max}\approx K\frac{1}{1+BAR}\left( \frac{1}{2}P_{gmax}- \left( R_{max}+RRR*BAR \right) \right),$$

$$\therefore K\approx\frac{\mu_{max}\left( 1+BAR \right)}{\frac{1}{2}P_{gmax}- \left( R_{max}+RRR*BAR \right)}.$$

Thus, since our fitted *P_gmax_* = 11.64 mg 0_2_ g^-1^ ABG DW h^-1^, *R_max_* = 1.987 mg 0_2_ g^-1^ ABG DW h^-1^, the roots and rhizomes respiration [RRR] = *R_max_*/10, the below-ground to above-ground biomass ratio [BAR] was set to 1 and the greatest value of μ_max_ we found in the literature was 0.001958333 h^-1^ (Nielsen and Pedersen, 2000; Zharova et al., 2008), the calculated value for K was set to 0.001075 g total DW mg^-1^ O_2_.

**S1.4 Mortality parameters**

For *C. nodosa* mortality rates we considered two potential processes: one where clonal integration is absent (no self-facilitation); and a second where this mechanism is present, producing a self-facilitation that decreases the mortality rates of the seagrass (Mayol et al., 2022). These two processes, together with the choice of including the presence or absence of photoacclimation, results in the four models tested in the present work.

For meadows where self-facilitation is absent, we assumed the values reported by Mascaró et al. (2014). In that study, they reported the values of mortality per month, as well as an integrated mortality rate for one year in two seagrass meadows of *C. nodosa*. We used the mortality rate values from the oligotrophic meadow to avoid the interaction with algal overgrowth (increased mortality). We then selected the values of July, since it was the month in which the photosynthetic measures by Marín-Guirao et al. (2022) were obtained. Therefore, we set *d_0_* = 0.000116 h^-1^, as the constant for the mortality rate which is always proportional to the current biomass of seagrass, for meadows where self-facilitation is absent.

For meadows where self-facilitation is present, we assumed an increased mortality (*δ*) for low levels of biomass and a nonlinear relationship that reduces *δ* as biomass levels increase. Thus, we modelled this self-facilitation as a sigmoidal function that yields an increased mortality rate for low levels of biomass. However, as biomass in the system increases, mortality rates start to decrease with a slope *λ_B_* after crossing a threshold (*B_0_* = 10), leading to very low values of seagrass mortality when biomass levels are high (Mayol et al., 2022).

**References**

Collier, C. J., Ow, Y. X., Langlois, L., Uthicke, S., Johansson, C. L., O’Brien, K. R., … Adams, M. P. (2017). Optimum Temperatures for Net Primary Productivity of Three Tropical Seagrass Species. *Frontiers in Plant Science*, 8. doi: 10.3389/fpls.2017.01446

Marín-Guirao, L., Bernardeau-Esteller, J., Belando, M. D., García-Muñoz, R., Ramos-Segura, A., Alcoverro, T., … Ruiz, J. M. (2022). Photo-acclimatory thresholds anticipate sudden shifts in seagrass ecosystem state under reduced light conditions. *Marine Environmental Research*, 177, 105636. doi: 10.1016/j.marenvres.2022.105636

Méndez, C. M. (2015). Factors driving herbivores consumption and feeding preferences across different macrophytes ecosystems (Doctoral dissertation, Universitat d'Alacant-Universidad de Alicante).

Mascaró, O., Romero, J., and Pérez, M. (2014). Seasonal uncoupling of demographic processes in a marine clonal plant. *Estuarine, Coastal and Shelf Science*, 142, 23–31. doi: 10.1016/j.ecss.2014.03.011

Nielsen, S. L., and Pedersen, M. F. (2000). Growth, photosynthesis and nutrient content of seedlings and mature plants of *Cymodocea nodosa* — the importance of clonal integration. *Aquatic Botany*, *68*(3), 265–271. doi: 10.1016/S0304-3770(00)00121-2

Pérez, M., and J. Romero. (1992). Photosynthetic response to light and temperature of the seagrass Cymodocea nodosa and the prediction of its seasonality. *Aquat. Bot*. 43, 51–62. doi:10.1016/0304-3770(92)90013-9

Pérez, M., Duarte, C. M., Romero, J., Sand-Jensen, K., and Alcoverro, T. (1994). Growth plasticity in *Cymodocea nodosa* stands: the importance of nutrient supply. *Aquatic Botany*, 47(3–4), 249–264. doi: 10.1016/0304-3770(94)90056-6

Staehr, P. A., and Borum, J. (2011). Seasonal acclimation in metabolism reduces light requirements of eelgrass (*Zostera marina*). *Journal of Experimental Marine Biology and Ecology*, 407(2), 139–146. doi: 10.1016/j.jembe.2011.05.031

Vonk, J. A., Christianen, M. J., Stapel, J., and O’Brien, K. R. (2015). What lies beneath: why knowledge of belowground biomass dynamics is crucial to effective seagrass management. *Ecological Indicators*, 57, 259-267.

Zharova, N., Sfriso, A., Pavoni, B., and Voinov, A. (2008). Analysis of annual fluctuations of *C. nodosa* in the Venice lagoon: Modeling approach. *Ecological Modelling*, 216(2), 134–144. doi: 10.1016/j.ecolmodel.2008.03.001

# Supplementary Figures and Tables

| **Table S1.** Values and standard errors (SE) for the photo-physiological response of *Cymodocea nodosa* after two months of experimental field shading (light reduction values as instantaneous irradiance in μmol quanta m^-2^ s^-1^ and in percentage of surface irradiance). Photosynthetic-Irradiance (*P-I*) parameters: maximum gross photosynthesis (*P_max_*) and above-ground respiration (*R*) are shown after conversion from μmol O_2_ g^-1^ ABG FW h^-1^ to mg O_2_ ABG DW h^-1^; and saturation irradiance (*I_k_*) is shown in units of μmol quanta m^-2^ s^-1^. | | | | | | | |
| --- | --- | --- | --- | --- | --- | --- | --- |
| **Ins. irradiance (μmol quanta m^-2^ s^-1^)** | **Surface irradiance (% SI)** | ***P_max_*** | ***P_max_* (SE)** | ***R*** | ***R* (SE)** | ***I_k_*** | ***I_k_* (SE)** |
| 634,96 | 74 | 11,2110684 | 0,44938581 | 1,97954554 | 0,0451396 | 67,6174237 | 7,12340965 |
| 514,82 | 60 | 12,0879902 | 0,60707836 | 1,93366887 | 0,01490785 | 71,5354374 | 8,4700463 |
| 360,42 | 42 | 11,1221678 | 0,63719271 | 2,04638972 | 0,00388821 | 54,3873511 | 7,30750426 |
| 274,54 | 32 | 11,9533882 | 0,56001197 | 1,9710401 | 0,0907847 | 70,075462 | 9,88600491 |
| 223,14 | 26 | 9,35382663 | 0,81513649 | 1,3734172 | 0,05134231 | 47,6515868 | 4,36793889 |
| 214,58 | 25 | 10,9309355 | 1,09412616 | 1,67061898 | 0,13280724 | 43,9724649 | 3,88216705 |
| 137,26 | 16 | 10,8572891 | 0,61362881 | 1,58959835 | 0,05436356 | 46,8406129 | 9,25578237 |
| 85,88 | 10 | 8,91133928 | 1,53433285 | 1,43301247 | 0,13259262 | 38,5555954 | 6,81642059 |
| 34,26 | 4 | 8,5697564 | 0,72797163 | 1,47591874 | 0,21613532 | 34,3856763 | 3,06302302 |

| **Table S2.** Summary of nonlinear fits of the physiological photoacclimation models (left panels in Fig. 1) of *Cymodocea nodosa* against light reduction. The values of each estimated parameter are shown in Table 1. | | | | | |
| --- | --- | --- | --- | --- | --- |
| Non-linear fitting of P_gross_ (I) | | | | | |
|  | Estimate | Std. Error | t value | Pr (> \|t\|) | (*) |
| P_gmax_ | 11.64 | 0.8837 | 13.174 | 4.5 e^-05^ | *** |
| P_gmin_ | 2.096 | >> Estimate | 0.028 | 0.979 |  |
| λ_p_ | 0.008524 | 0.0195 | 0.435 | 0.681 |  |
| y_cP_ | -49.1 | >> Estimate | -0.032 | 0.975 |  |
| Residual standard error: 0.9227 on 5 degrees of freedom | | | | | |
| Number of iterations to convergence = 17 | | | | | |
| Achieved convergence tolerance: 1.49 e^-08^ | | | | | |
| Non-linear fitting of R (I) | | | | | |
|  | Estimate | Std. Error | t value | Pr (> \|t\|) | (*) |
| R_max_ | 1.987 | 0.06547 | 30.34 | 7.29 e^-07^ | *** |
| R_min_ | 1.509 | 0.05670 | 26.61 | 1.40 e^-06^ | *** |
| λ_R_ | 0.4567 | >> Estimate | 0.00 | 1 |  |
| y_cR_ | 267.1 | >> Estimate | 0.00 | 1 |  |
| Residual standard error: 0.1134 on 5 degrees of freedom | | | | | |
| Number of iterations to convergence = 35 | | | | | |
| Achieved convergence tolerance: 1.49 e^-08^ | | | | | |
| Non-linear fitting of I_k_ (I) | | | | | |
|  | Estimate | Std. Error | t value | Pr (> \|t\|) | (*) |
| I_kmax_ | 70.04809 | 10.53706 | 6.648 | 0.00116 | ** |
| I_kmin_ | 24.54396 | 49.72699 | 0.494 | 0.64252 |  |
| λ_K_ | 0.00863 | 0.01362 | 0.634 | 0.55417 |  |
| y_cK_ | 177.45783 | 259.00230 | 0.685 | 0.52371 |  |
| Residual standard error: 8.315 on 5 degrees of freedom | | | | | |
| Number of iterations to convergence = 19 | | | | | |
| Achieved convergence tolerance: 1.49 e^-08^ | | | | | |
